# A Case Study on Choosing Normalization Methods and Test Statistics for Two-Channel Microarray Data

**DOI:** 10.1002/cfg.416

**Published:** 2004-07

**Authors:** Yang Xie, Kyeong S. Jeong, Wei Pan, Arkady Khodursky, Bradley P. Carlin

**Affiliations:** 1 Division of Biostatistics MMC 303 School of Public Health University of Minnesota Minneapolis MN 55455-0392 USA; 2 Department of Biochemistry Molecular Biology and Biophysics University of Minnesota Minneapolis MN USA

## Abstract

DNA microarray analysis is a biological technology which permits the whole genome to be monitored simultaneously on a single slide. Microarray technology not only opens an exciting research area for biologists, but also provides significant new challenges to statisticians. Two very common questions in the analysis of microarray data are, first, should we normalize arrays to remove potential systematic biases, and if so, what normalization method should we use? Second, how should we then implement tests of statistical significance? Straightforward and uniform answers to these questions remain elusive. In this paper, we use a real data example to illustrate a practical approach to addressing these questions. Our data is taken from a DNA–protein binding microarray experiment aimed at furthering our understanding of transcription regulation mechanisms, one of the most important issues in biology. For the purpose of preprocessing data, we suggest looking at descriptive plots first to decide whether we need preliminary normalization and, if so, how this should be accomplished. For subsequent comparative inference, we recommend use of an empirical Bayes method (the *B* statistic), since it performs much better than traditional methods, such as the sample mean (*M* statistic) and Student's *t* statistic, and it is also relatively easy to compute and explain compared to the others. The false discovery rate (FDR) is used to evaluate the different methods, and our comparative results lend support to our above suggestions.


**Comparative and Functional Genomics**
*Comp Funct Genom* 2004; **5**: 432–444.
Published online in Wiley InterScience (www.interscience.wiley.com). **DOI:** 10.1002/cfg.416

**Research Article**

### A case study on choosing normalization methods and test statistics for two-channel microarray data

Yang Xie[1], Kyeong S. Jeong[2], Wei Pan[1], Arkady Khodursky[2] and Bradley P. Carlin[1]*
[1] *Division of Biostatistics, School of Public Health, University of Minnesota, Minneapolis, MN, USA*
[2] *Department of Biochemistry, Molecular Biology and Biophysics, University of Minnesota, Minneapolis, MN, USA*

**Correspondence to: Bradley P. Carlin, Division of Biostatistics, MMC 303, School of Public Health, University of Minnesota, Minneapolis, MN 55455-0392, USA. E-mail: brad@biostat.umn.edu*

Received: 4 December 2003
Revised: 28 May 2004
Accepted: 18 June 2004

### Abstract

**DNA microarray analysis is a biological technology which permits the whole genome to be monitored simultaneously on a single slide. Microarray technology not only opens an exciting research area for biologists, but also provides significant new challenges to statisticians. Two very common questions in the analysis of microarray data are, first, should we normalize arrays to remove potential systematic biases, and if so, what normalization method should we use? Second, how should we then implement tests of statistical significance? Straightforward and uniform answers to these questions remain elusive. In this paper, we use a real data example to illustrate a practical approach to addressing these questions. Our data is taken from a DNA–protein binding microarray experiment aimed at furthering our understanding of transcription regulation mechanisms, one of the most important issues in biology. For the purpose of preprocessing data, we suggest looking at descriptive plots first to decide whether we need preliminary normalization and, if so, how this should be accomplished. For subsequent comparative inference, we recommend use of an empirical Bayes method (the *B* statistic), since it performs much better than traditional methods, such as the sample mean (*M* statistic) and Student's *t* statistic, and it is also relatively easy to compute and explain compared to the others. The false discovery rate (FDR) is used to evaluate the different methods, and our comparative results lend support to our above suggestions. Copyright © 2004 John Wiley & Sons, Ltd.**

**Keywords: empirical Bayes methods; normalization; false discovery rate; significance testing; spatial effects; background correction**

### Introduction

### Biological background

Gene expression is a process of 'the full use of the information in a gene via transcription and translation, leading to production of a protein and hence the appearance of the phenotype determined by that gene' (Lackie and Dow, 1999). The gene expression process determines the intracellular concentration of proteins, which play an important role in many biological systems. On the other hand, the gene expression procedure is controlled by certain proteins (regulators) in an organized way. As a result, the knowledge of gene expression and transcription regulation are two key questions in biology. Answers to these questions will facilitate basic biology and medical research, leading to applications in clinical diagnosis, disease classification and finding new treatments for diseases.

DNA microarray experimentation is a biological technology which permits the whole genome to be monitored on a single slide, so that a better picture of the interaction among thousands of genes can be observed simultaneously (Brazma *et al.*, 2000). As a result of microarray applications, the research

Copyright © 2004 John Wiley & Sons, Ltd.

focus of biologists has shifted from individual genes to multiple genes, and their interaction and cooperation in a complicated way to maintain life. One important microarray application is to compare the expression levels of genes in samples drawn from different tissues or conditions through a transcriptional profiling experiment (Tani *et al.*, 2002; Spellman *et al.*, 1998). Another ingenious application of microarray technology is to find the DNA-binding sites for proteins across the entire genome through a chromatin immunoprecipitation (CHIP) experiment (Ren *et al.*, 2000; Iyer *et al.*, 2001), where the interest is to detect the target DNA sequences that are bound by specific proteins.

### Statistical issues

Microarray technology not only opens an exciting research area for biologists, it also provides significant challenges to statisticians. A common and difficult question before starting any data analysis is whether we need to preprocess the data, and if so how. Generally, there are some systematic biases and variations in microarray experiments, such as label or dye effects and slide or spatial effects, which may affect the measurements of gene expression levels, and thus the conclusion of an experiment (Yang *et al.*, 2002; Quackenbush, 2002). In order to remove these biases and make the multiple arrays comparable, various normalization methods have been proposed. For a chromatin immunoprecipitation experiment using genomic DNA as control samples, intensities of the control genomic DNA samples should be around a constant, while for a transcriptional profiling experiment, the intensities in the reference channel will have gene-specific means. This different property may require different normalization methods. This paper briefly describes several normalization methods and aims to provide a practical approach to decision making in this regard. We will illustrate our approach for both chromatin immunoprecipitation and transcriptional profiling experiments.

Another challenging question is how to properly carry out statistical data analysis. There are several overriding statistical issues here. One has to do with the 'large $p$, small $n$ paradigm' (West, 2000). Thousands of genes will be tested at the same time, but generally we only have a few replications of each. Empirical Bayes (EB) methods provide a natural approach to addressing this problem because they can effectively borrow information across genes (Efron and Morris, 1973; Carlin and Louis, 2000), and so EB methods for microarray have recently been implemented by a variety of researchers (Newton *et al.*, 2001, 2003, 2004; Lonnstedt and Speed, 2002; Kendziorski *et al.*, 2003; Lin *et al.*, 2003). A relevant statistical issue is how to determine the statistical significance for testing a null hypothesis. In many situations, it is not straightforward to obtain the null distribution of a chosen test statistic, so it is not easy to determine the cut-off for rejection region or $p$ value for the null hypothesis. However, because microarray experiments are largely exploratory in nature, investigators generally care little about precise $p$ values, and are willing to accept some false positives among the identified genes. Thus, the best method is one that can lower both type I and type II errors over a range of cut-offs (Lonnstedt and Speed, 2002). This paper considers four traditional or new test statistics used for microarray data analysis, especially focusing on one EB method, and compares the methods based on plots and their false discovery rates.

This paper is organized as follows. 'Data and Methods' contains a brief description of the dataset used in the paper, as well as the various normalization methods, test statistics, evaluation methods and their implementation. The 'Results' section then illustrates in the context of our microarray experiment; in particular, firstly, we show the effects of normalization methods on the location and scale of the intensity measurements. In the following subsection plots are used to illustrate the utility of an empirical Bayes method and how it differs from other methods. After that we compare the results of using different normalization methods and statistics by looking at their overlap rates of identified genes and their false discovery rates. In the last subsection, we apply our proposed approach to a transcriptional profiling experiment. Finally, there is a discussion of our findings and open questions for future investigation.

### Data and methods

### Data

Data from a study of transcription regulation mechanisms is used in this project. The overall goal of

Copyright © 2004 John Wiley & Sons, Ltd. 

the study is to find the DNA binding sites *in vivo* of a broad transcription regulator, leucine responsive regulatory protein (Lrp) (Tani *et al.*, 2002), through analysing the genome-wide distribution of Lrp. Detailed descriptions of the method of genome-wide location and function of DNA binding proteins can be found elsewhere (Ren *et al.*, 2000; Iyer *et al.*, 2001).

Briefly, to identify the target binding sites of Lrp, the combination of chromatin immunoprecipitation and microarray hybridization was used. DNA samples from wild-type *Escherichia coli* were labelled with red (Cy5) fluorophore following cross-linking, immunoprecipitation and amplification; we call these samples 'test samples'. Genomic DNA samples were also prepared and labelled with green (Cy3) fluorophore; we call these samples as 'control samples'. We identify the genomic target loci by comparative hybridization of test and control samples to a DNA microarray. The ratio of Cy5 to Cy3 fluorescence intensities measured at each spot in the microarray provided a measure of the extent of binding of Lrp to the corresponding genomic locus. If there is no binding of Lrp, this ratio should be a constant across all of the genes. So the specific purpose of this microarray analysis is to identify the spots or genes with intensity ratios between the test samples and the control samples that are different from this constant. Each DNA microarray includes 4221 ORFs of *E. coli*. After dealing with missing data and background correction, 4011 genes with five replicated arrays are used to do the analysis. In this paper, we define $M_{ij}$ as the log ratio of the background-corrected intensity levels in test and control samples for gene $i$ on array $j$:

$$M_{ij} = \log_2 \frac{R_{ij}}{G_{ij}} = \log_2 \frac{(\text{intensity level for test sample})_{ij}}{(\text{intensity level for control sample})_{ij}}$$

for $i = 1, \ldots, N$ and $j = 1, \ldots, n$, where $N = 4011$ and $n = 5$ in this experiment. Suppose that $\mu_i$ and $M_i$ are the population and sample means of the $M_{ij}$, respectively. Our goal is to test the null hypothesis $H_0{:}\mu_i = c$ for each gene $i$, where c is a constant across all of the genes. We can estimate this constant by the median of the $M_{ij}$ for each array, which we call $\hat{c}_j$. If we transform each $M_{ij}$ by subtracting $\hat{c}_j$, our null hypothesis becomes $H_0{:}\mu_i = 0$ for each gene $i$. For this data set, we apply this transformation before any further analysis, and do not regard this as a normalization method.

We also define $A_{ij}$ as the log of geometric mean of the two channel intensities; that is, $A_{ij} = (\log_2 R_{ij} + \log_2 G_{ij})/2$. We denote the sample mean of $A_{ij}$ by $A_i$ and will use generic notation $M$ and $A$ for $M_i$ and $A_i$.

### Normalization methods

In microarray experiments, the purpose of normalization is to remove the systematic variation, such as the differences in labelling efficiency between two fluorescent dyes used. There are various sources of biases, including experimental variability in the processing procedures and the scanner settings at the data collection step. Some of these factors lead to biases that depend on the spot's intensity or its location on the array, often referred to as spatial or 'print-tip' effects. Locally weighted smoother (lowess) normalization and print-tip group lowess normalization methods were proposed to correct these kinds of biases (Yang *et al.*, 2002). The lowess normalization method is a within-slide location normalization method. It assumes that the (dye) bias depends on the spot intensity, so it adjusts the log-ratios $M_{ij}$ by an intensity-dependent mean curve $c(A)$, the lowess fit in a scatterplot of the log-ratio $M$ vs. overall spot intensity $A$. The print-tip group lowess normalization method assumes that there are systematic differences between the print-tips, so it adjusts the log-ratios by both the intensity and print-tip effects $c_k(A)$, the lowess fit in an $M$ vs. $A$ plot for the $k$th grid only. Both of these methods are used after a summary measurement of gene intensity level [typically $log(R/G)$] is obtained for two-channel arrays. Normalization can be applied for the purpose of constructing an expression value using physical and biological properties, as well as for standardizing expression value for within- and between-sample variability. The purpose of our normalization is the latter. Irizarry *et al.* (2003) address some of the normalization issues in a coherent way.

The preceding discussion notwithstanding, the questions of whether and how to normalize the data do arise in practice. We address these questions by looking at some descriptive plots. First, a

Copyright © 2004 John Wiley & Sons, Ltd.

scatterplot of $M$ vs. $A$ is checked for each slide to see whether there is any systematic pattern between the log-ratio intensity and the overall intensity; if there is, we may need to do normalization. Then within print-tip lowess curves are fitted for each print-tip group and the spatial plots are checked to see whether there are spatial effects. If the lowess curves are different for each print-tip group and the intensities are disproportionately distributed among the print-tip groups, then we may need to consider the print-tip group lowess normalization.

### Test statistics

In this project, we use four different statistical methods to analyse the data. The first one is the $M$-statistic, $M_i$, which corresponds to the early practice of simply using twofold change as a significance indicator. $M$ does not take account of possibly different variations of $M_{ij}$ for different genes, and effectively it treats a highly variable gene in the same way as a stable one. A second possibility is the Student's $t$ statistic, $t_i = M_i/SE_i$, where $SE_i$ is the estimated standard error of $M_i$. The $t_i$ statistic can be regarded as a standardized version of $M_i$.

Because we have thousands of genes but only a small number of replicates for each gene, it is quite possible that for some genes, just by chance, their $SE$ estimates (based on sample variances) can be very small, leading to a huge $t$ statistic. In order to address this problem, another statistic, $S$, was proposed by Tusher *et al.* (2001). This statistic is a modified $t$ statistic that adds a constant $a_0$ into the denominator, i.e. $S_i = M_i/(SE_i + a_0)$. As suggested by Tusher *et al.* (2001), we use the median of standard errors of all the genes as $a_0$. The motivation of the $S$-statistic is intuitive, but it does not have a rigorous justification (although a connection exists between the $S$-statistic and a Bayesian-regularized $t$ statistic; Baldi and Long, 2001).

The last method we consider is the $B$ statistic (Lonnstedt and Speed, 2002), which is an empirical Bayes estimate of the log posterior odds of $\mu_i \neq 0$. We assume that genes are independent and the measurement $M_{ij}$ is a random variable from a normal distribution with mean $\mu_i$ and variance $\sigma_i^2$:

$$M_{ij}|\mu_i, \sigma_i \sim N(\mu_i, \sigma_i^2)$$

Most genes have the same mean intensity level between the two samples, corresponding to $\mu_i = 0$. Only a small proportion (say, $p$) of genes have different mean intensities, leading to $\mu_i \neq 0$. An indicator function $\Delta_i$ is defined as 0 if $\mu_i = 0$ and as 1 if $\mu_i \neq 0$. By definition and Bayes rule, we can calculate the log posterior odds for gene $i$ having $\Delta_i = 1$:

$$B_i = \log \frac{Pr(\Delta_i = 1|\{M_{ij}\})}{Pr(\Delta_i = 0|\{M_{ij}\})} = \log \frac{p \prod_{i=1}^{n} Pr(M_{ij}|\Delta_i = 1)}{(1-p)\prod_{i=1}^{n} Pr(M_{ij}|\Delta_i = 0)}$$

We use conjugate prior distributions for mean $\mu_i$ and variance $\sigma_i^2$. For $n$ arrays, a degrees of freedom parameter $v$, and scale parameters $a > 0$ and $c > 0$, we set $\tau_i = na/2\sigma_i^2$ and assume that $\tau_i \sim Gamma(v, 1)$, and:

$$\mu_i|\tau_i \begin{cases} \sim N(0, cna/2\tau_i) & \text{if } \Delta_i = 1 \\ = 0 & \text{if } \Delta_i = 0 \end{cases} \quad (1)$$

Because this is a conjugate prior, we can easily calculate the joint distribution of $\{M_{ij}, j = 1, \ldots, n\}$, $\mu_i$, and $\tau_i$, and then integrate to get the marginal distributions of $Pr(M_{ij}|\Delta_i = 1)$ and $Pr(M_{ij}|\Delta_i = 0)$. The final expression for $B$ is then:

$$B_i = \log \frac{p}{1-p} \frac{1}{\sqrt{(1+nc)}} \times \left[ \frac{a + s_i^2 + M_i^2}{a + s_i^2 + \dfrac{M_i^2}{1+nc}} \right]^{v+\frac{n}{2}}$$

where $s_i^2$ is the sum of squared errors over $n$ arrays for gene $i$. From this formula, we can find that the only gene-specific part lies in the last ratio, which is always greater or equal to 1, since $1 + nc \geq 1$. Thus, we can deduce a monotonically increasing relationship between $B_i$ and $M_i^2$ or relative gene intensity levels, and the relationship is stronger if the variance for the gene is smaller. (A similar relationship exists between $t_i$ and $M_i$.)

$B$ has four hyperparameters: $p$, $v$, $a$ and $c$. Since there are no consistent estimates for them and appropriate hyperpriors are not clear, we use

Copyright © 2004 John Wiley & Sons, Ltd.

an EB approach to estimate them. First, we fix $p$ and estimate $v$, $a$ and $c$. The methods of moments is used to get $\widehat{a}$ and $\widehat{v}$, and the least squares method to get $\widehat{c}$. There are no satisfactory estimates for $p$, but in most of cases that will not affect the shape of the $B$ vs. $M$ plot (Lonnstedt and Speed, 2002).

### Evaluation methods

Three plots with numbers indicating whether the genes are identified as being bound to Lrp by different statistics are used to compare the false positive and false negative rates among the $M$, $t$ and $B$ statistics: average $M$ vs. variance of $M$, $t$ vs. average intensity $A$, and $B$ vs. $M$. The overlap rates of top genes identified by different normalization methods and statistics are calculated to indicate the agreement between two different methods. The higher the overlap rate between two methods, the better agreement between them. We also use the false discovery rate (Efron *et al.*, 2001; Benjamini and Hochberg, 1995) to compare the three normalization methods and the four statistics. FDR is an alternative to controlling the false positive rate (type I error), and is defined as the expected proportion of false positive genes (FP) among total positive genes (TP); the observed FP : TP ratio is often used to estimate FDR. When we use FDR to compare various statistical methods, we prefer the method that gives the lowest FDR while giving the same number of top (i.e. positive) genes as that of all other methods.

### Implementation

We implemented the methods in the R software package (**www.r-project.org**). In particular, we used the SMA (Statistics for Microarray Analysis) package developed by Dudoit *et al.* (2002) (**stat-www.berkeley.edu/users/sandrine/software.html**). We used SMA to do lowess normalization and print-tip group normalization, creating the $M$ vs. $A$ plots, boxplots and spatial plots, and calculating $B$ statistics.

To calculate FDR, we used a permutation method to estimate the false positive number FP (Tusher *et al.*, 2001). Under the null hypothesis, $H_0$:$\mu_i = 0$, we can generate a permuted dataset as follows: multiplying each $M_{ij}$ by either 1 or −1 randomly. For example, suppose that the original parameters $M_{ij}$ for gene $i$ are: 0.2, 0.4, −0.3, −0.5, 0.1. We generated five random numbers, say, −1, 1, −1, 1, −1. Then our permuted data will be: −0.2, 0.4, 0.3, −0.5, −0.1. We do this permutation 50 times for each genes. The false positive number from each permutation is the number of genes that counted as significant genes from the permuted data. The average of the false positive numbers over 50 permutations is calculated as FP. The number of genes that counted as significant genes from the original data is regarded as TP, and we estimate FDR = FP/TP (Pan, 2003). Note that a more elaborate estimator of FDR, namely $\widehat{FDR} = \pi_0 FP/TP$, with $\pi_0$ as the prior probability of null hypothesis being true, has been proposed (Storey and Tibshirani, 2003). Since $\pi_0$ is a constant, independent of whatever normalization or test statistic is used, using this estimator will not influence our final results.

### Results

### Effects of normalization

$M$ vs. $A$ plots, spatial plots, and boxplots of the measurements in the first slide are displayed to compare the within-slide normalization methods; the corresponding plots for the other four slides are similar. Figure 1 shows that, before normalization, the intensity ratio increases as the average intensity increases, possibly indicating a systematic pattern. After the lowess normalization, this pattern disappears. Figure 2 displays 16 within-print-tip lowess lines, one for each print-tip group. This plot may indicate the existence of spatial effects, since six lowess curves seem to stand out from each other.

Figure 3 shows a spatial plot. There are disproportionately large numbers of extreme log-ratios in the upper four grids if we do not use any normalization method, possibly indicating spatial effects for the experiment and the need for within-print-tip group lowess location normalization. Finally, the within-print-tip boxplots in Figure 4 also indicate that both the mean and variances are different among the 16 print-tip groups. After within-print-tip group lowess normalization, the mean of log-ratio of each print-tip group is adjusted to zero. So we may conclude from these plots that there seem to be spatial effects and we need to use the print-tip group normalization.

Copyright © 2004 John Wiley & Sons, Ltd. 

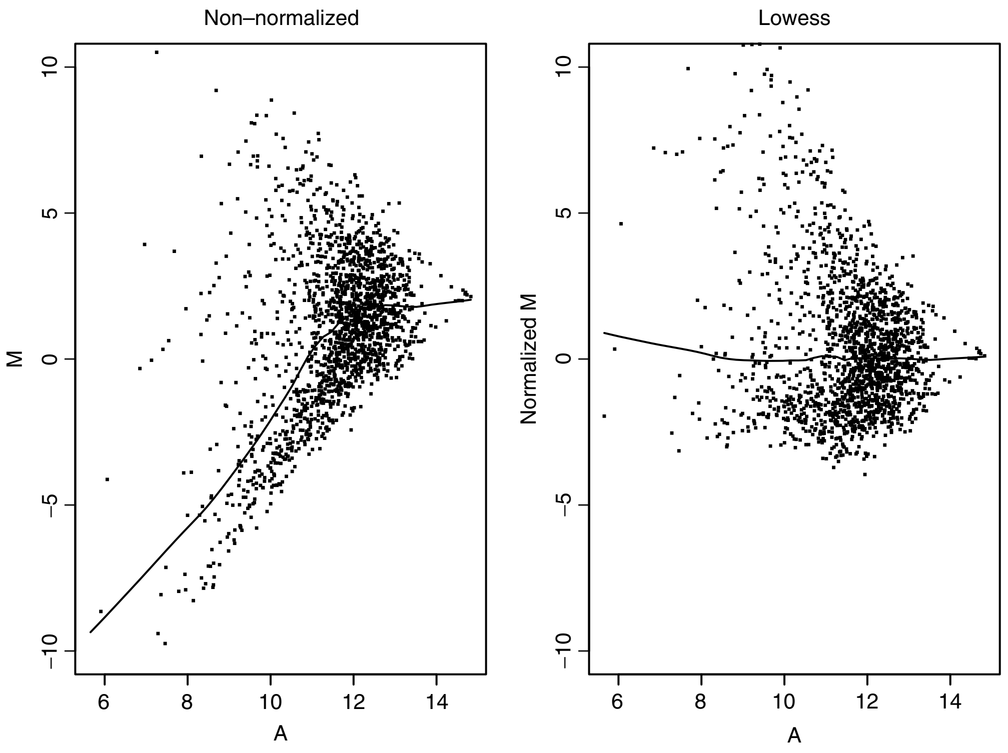

**Figure 1.** M vs. A plots under (a) no normalization and (b) lowess normalization for slide 1 with lowess curves superimposed

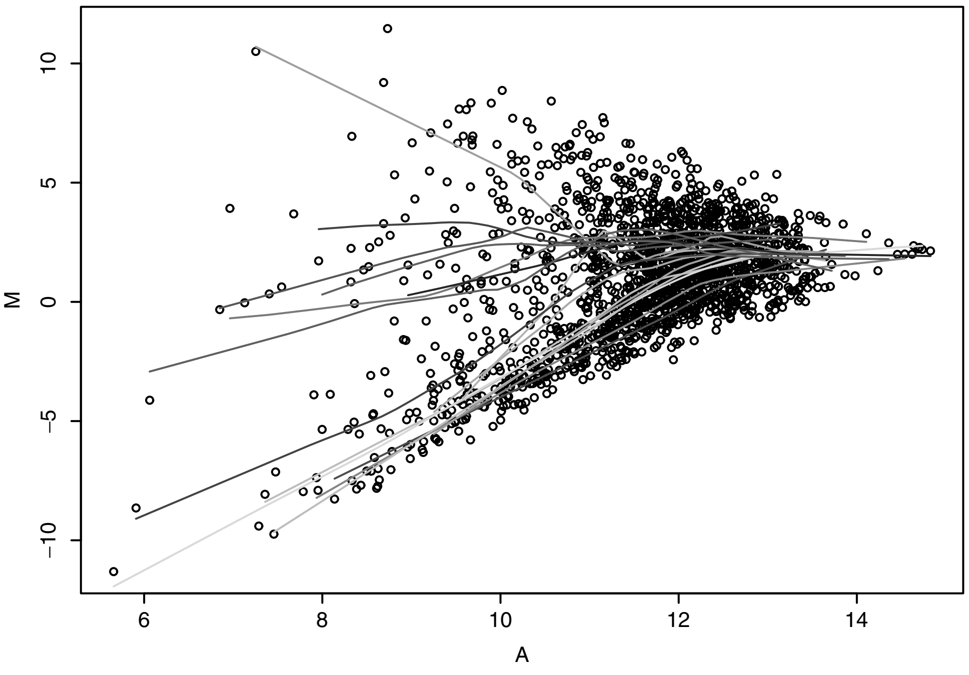

**Figure 2.** *M* vs. *A* plot with 16 individual lowess smoothing lines superimposed, one for each print-tip group in slide 1

Copyright © 2004 John Wiley & Sons, Ltd. 

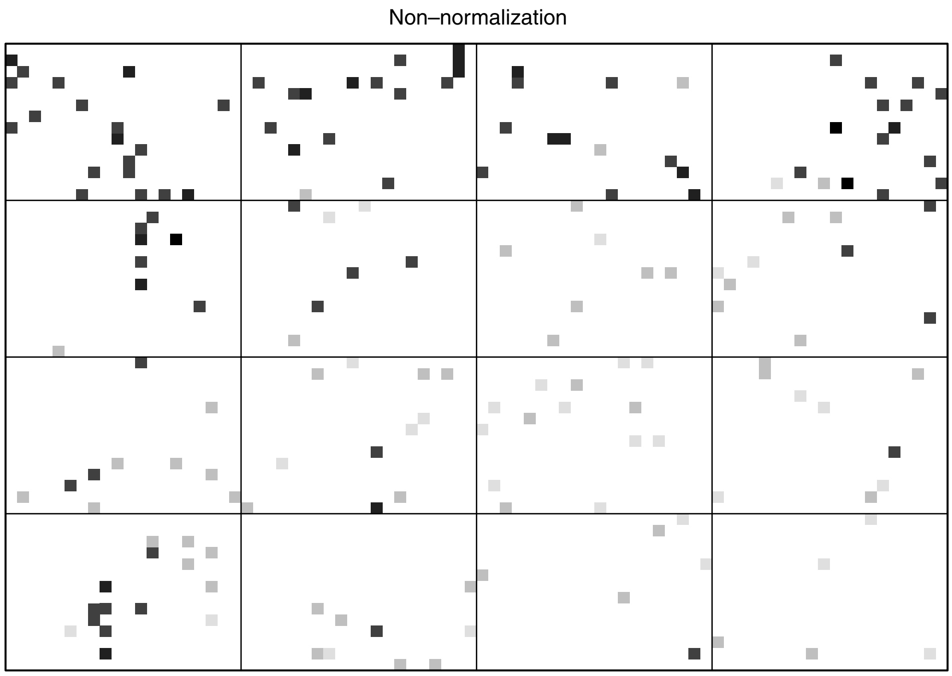

**Figure 3.** Spatial plots of slide 1, highlighting the spots with 5% extreme log-ratio intensities prior to normalization. Each rectangular represents the log-ratio of a spot on the array. The dark rectangular represents positive log-ratios and the light rectangular represents negative log-ratios

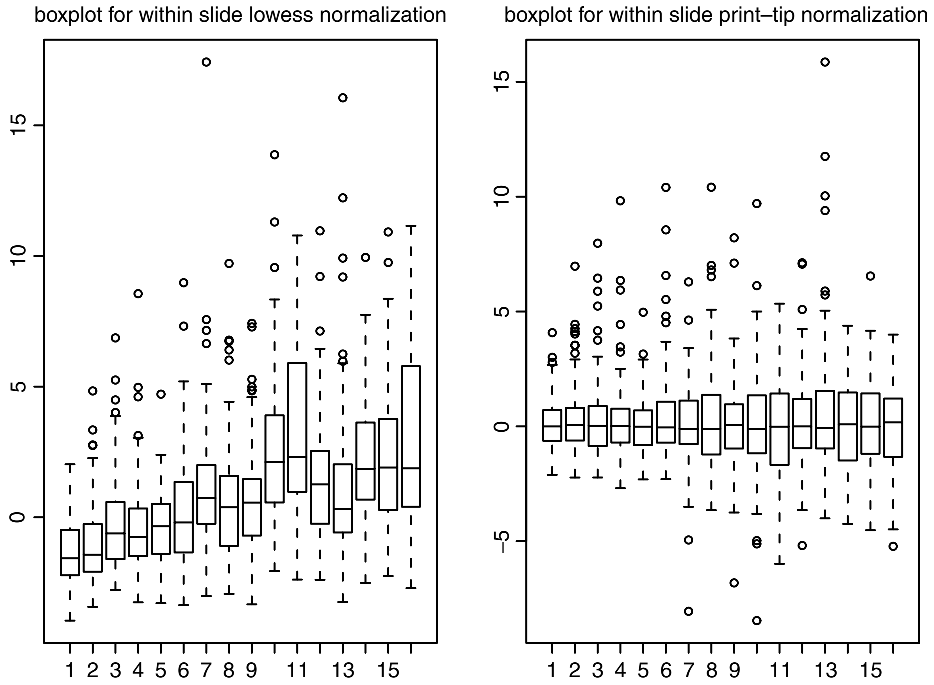

**Figure 4.** Boxplots displaying (a) the log-ratio distribution after lowess normalization, and (b) within-print-tip group lowess normalization for each of the 16 print-tip groups. The array was printed using a 4 × 4 set of print-tips

Copyright © 2004 John Wiley & Sons, Ltd. 

### Effects of using various statistics

In this subsection, we illustrate the utility of $B$ and how it differs from $M$ and $t$, based on plots following the idea of Lonnstedt and Speed (2002). Corresponding illustration of the $S$ statistic can be found elsewhere (Tusher *et al.*, 2001; Efron *et al.*, 2000).

All statistics were calculated after applying the print-tip group normalization method to the data. The various plots relating the statistics $M$, $t$ and $B$ are shown in Figure 5. Genes that are identified as 'extreme' by at least one of these three statistics are plotted not as dots but as numbers in this figure; Table 1 provides the key to identifying to which of the $2^3 - 1 = 7$ possible sets these extreme genes belong. We selected the cut-off points so that each statistic will identify its top 150 genes as being extreme. Specifically, the genes are selected as extreme for $M$ if $|M_i| > 1.43$, for $t$ if $|t_i| > 4.62$, and for $B$ if $B_i > -2.11$. After setting these cut-off points, all of the seven possible sets in Table 1 are non-empty except Set 6.

**Table 1.** Number of genes falling in the corresponding sets, 1–7. In columns 2–4, a '1' indicates that the genes in this set are 'extreme' for the given statistic

| | Extreme for | | | |
|---|---|---|---|---|
| **Set** | ***M*** | ***t*** | ***B*** | **Number of genes in set** |
| 1 | 0 | 0 | 1 | 48 |
| 2 | 0 | 1 | 0 | 91 |
| 3 | 0 | 1 | 1 | 41 |
| 4 | 1 | 0 | 0 | 89 |
| 5 | 1 | 0 | 1 | 43 |
| 6 | 1 | 1 | 0 | 0 |
| 7 | 1 | 1 | 1 | 17 |

From the $M$ mean vs. log variance plot in Figure 5, we see that at the left end of the plot, the

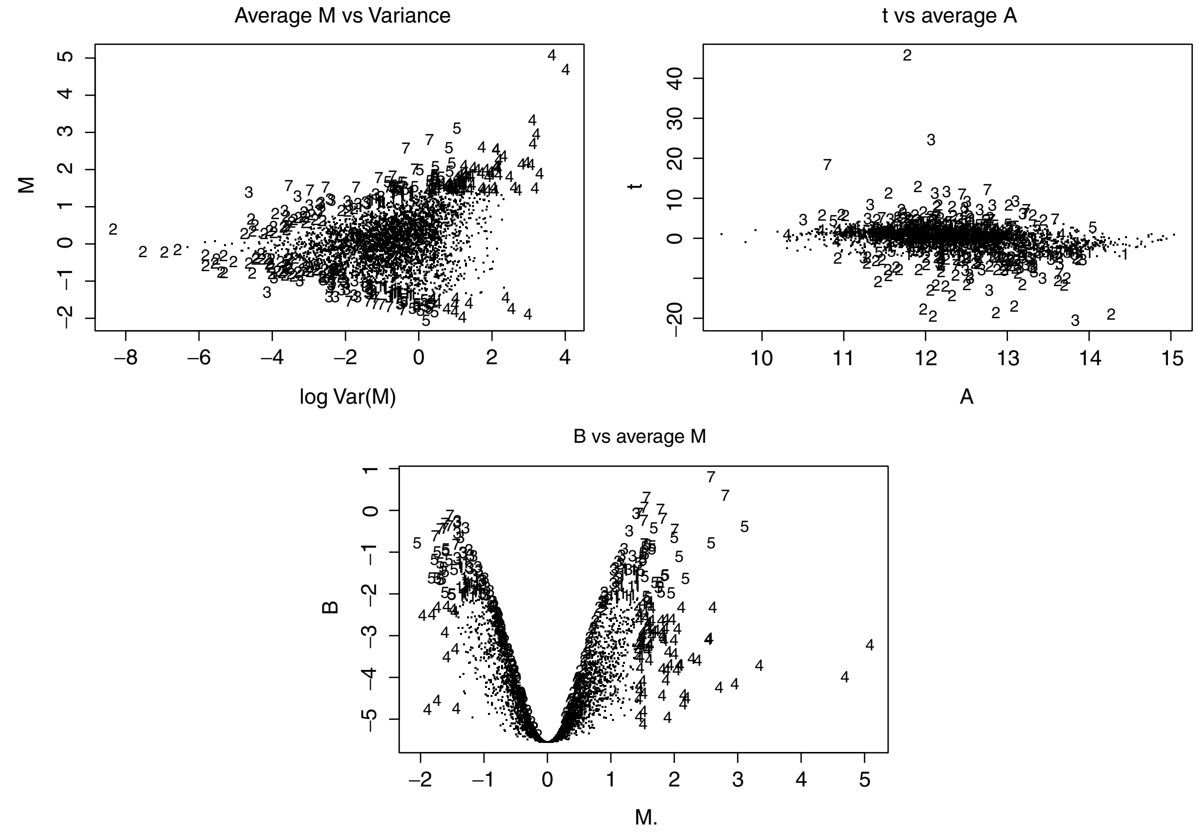

**Figure 5.** Lrp data for different statistics. The three plots are sample mean of $M$ vs. its sample variance, $t$ vs. average intensity $A$, and $B$ vs. $M$. When the plotting character is a number from 1 to 7, this indicates whether the gene is identified as extreme by $M$, $t$, $B$, or some combination thereof (see Table 1 for key)

Copyright © 2004 John Wiley & Sons, Ltd.

genes have very small variance and their means are not large. Almost all of these genes fall into Set 2; i.e. the genes are identified only by $t$ but not by $M$ and $B$ statistics, which is consistent with our previous description of $t$: it can be inflated by a small variance. It is likely that these genes are false positives from using the $t$ statistic.

It is reassuring to see that these genes are not identified by the $B$ statistic. When we look at the right end of the $M$ vs. log variance plot, some genes have a large mean but their variances are also large. All of these genes fall into Set 4; i.e. the genes are identified only by $M$ but not by $t$ and $B$. This phenomenon is consistent with our previous description of $M$: a large $M$ does not take account of its possibly large variation. We may consider these genes as false positives from using $M$.

The $t$ vs. $A$ plot indicates that most of the genes with extreme $t$ values fall into Set 2 and are not identified by $M$ and $B$, while the $B$ vs. $M$ plot shows that the genes with extreme $B$ values fall into Set 7; that is, these genes are identified by all of $M$, $t$ and $B$. Most of the genes falling in Set 1 that are identified only by $B$ have only moderately high $B$ values. Hence it appears that $B$ is more stable and reliable than $t$. Set 7 includes the genes with high values for all the three statistics, and this shows up clearly in the plots. Set 3 can be detected by $t$ and $B$, but not by $M$. Set 1 and Set 5 can be detected by $B$, but not by $t$. Set 6 is the set of genes that can be detected by $M$ and $t$ but not by $B$. We note that the number of genes falling into Sets 1, 3, and 5 are between 40 and 50, but there are no genes falling into Set 6, which confirms that the genes high in both $M$ and $t$ are also high in $B$.

### Evaluation of both normalization methods and test statistics

Table 2 shows the overlap rates of the top 150 genes identified by different statistics to compare the agreement among these statistics. The results are consistent across normalization method: the overlap rates among $M$, $t$ and $S$ statistics are below 50%, but there is more than an 80% overlap rate between $S$ and $B$. This appears related to the fact that both $B$ and $S$ can be justified from a Bayesian point of view.

Table 3 compares estimated FDRs for the genes identified as extreme by the four statistics before and after applying two normalization methods. The FDRs for non-normalized data are in the range 70–80% for the various statistics, while this range for the lowess normalization is 30–56%, and just 17–43% for the print-tip group normalization method. When we compare the FDRs for each test statistic using the same normalization method, we find that $M$ always has the highest FDR, and the FDR for $t$ is also rather high. By contrast, the $B$ statistic has the lowest FDR, with the $S$ statistic close behind. Since $B$ with print-tip normalization offers the lowest FDR (17%), we conclude that for this dataset, preprocessing to account for spatial effects and subsequent use of the $B$ statistic is the best approach.

### Application to transcription profiling data

To assess the performance and versatility of our approach, we also applied it to a transcription

**Table 3.** False discovery rates for the identified genes by statistic and normalization method

| | Normalization method | | |
|---|---|---|---|
| | **None** | **Lowess** | **Print-tip** |
| $M$ | 0.86 | 0.56 | 0.43 |
| $t$ | 0.71 | 0.43 | 0.35 |
| $S$ | 0.72 | 0.33 | 0.22 |
| $B$ | 0.69 | 0.30 | 0.17 |

**Table 2.** Overlap rates of the genes identified as extreme by the various statistics based on three different normalization methods

| | None | | | | Lowess | | | | Print-tip | | | |
|---|---|---|---|---|---|---|---|---|---|---|---|---|
| | **M** | **t** | **S** | **B** | **M** | **t** | **S** | **B** | **M** | **t** | **S** | **B** |
| $M$ | 1.00 | 0.15 | 0.43 | 0.46 | 1.00 | 0.07 | 0.31 | 0.33 | 1.00 | 0.11 | 0.39 | 0.40 |
| $t$ | | 1.00 | 0.41 | 0.38 | | 1.00 | 0.35 | 0.39 | | 1.00 | 0.42 | 0.39 |
| $S$ | | | 1.00 | 0.85 | | | 1.00 | 0.82 | | | 1.00 | 0.85 |
| $B$ | | | | 1.00 | | | | 1.00 | | | | 1.00 |

Copyright © 2004 John Wiley & Sons, Ltd. 

profiling dataset. Transcriptional profiling was carried out to comprehensively define a family of genes whose transcription depends on the activity of leucine-responsive regulatory protein, Lrp. Specifically, researchers set out to identify genes differentially expressed in $Lrp^+$ and $Lrp^-$ strains. Two-colour hybridization of the cDNA microarray, as described in Tani *et al.* (2002), was used in the experiment. Here we use six arrays of 4281 genes each to illustrate our proposed procedure of selecting normalization methods and test statistics.

Figure 6 displays 16 within-print-tip lowess lines for the Lrp expression data. From this plot, we can see that there is no obvious pattern between intensity ratio and average intensity, and the 16 lowess curves seem to stay close to each other, indicating that no spatial effects exist and hence any normalization (especially the within print-tip normalization) may not help much. Table 4 compares estimated FDRs for the top 150 genes identified by the four statistics before and after applying lowess and within print-tip normalization methods. As expected from Figure 6, the normalization does not have much effect on the FDR, and lowess normalization seems to be slightly better than no normalization and within-print-tip normalization. When we compare the four statistics, we find that $M$ always has the highest FDR and the other three statistics, $t$, $S$ and $B$, have much lower FDR, with $B$ having consistently the lowest FDR among these four. So, based on the plot and FDR, we conclude that for this dataset, normalization does not have much effect on results, and the $B$ statistic seems to offer the best approach.

**Table 4.** For Lrp expression data: false discovery rates for the identified genes by statistic and normalization method

| | Normalization method | | |
|---|---|---|---|
| | **None** | **Lowess** | **Print-tip** |
| $M$ | 0.171 | 0.162 | 0.169 |
| $t$ | 0.039 | 0.032 | 0.039 |
| $S$ | 0.031 | 0.032 | 0.031 |
| $B$ | 0.019 | 0.010 | 0.019 |

### Discussion

In this paper, we have compared three normalization methods and four test statistics to identify target genes bound by a protein called Lrp. Different

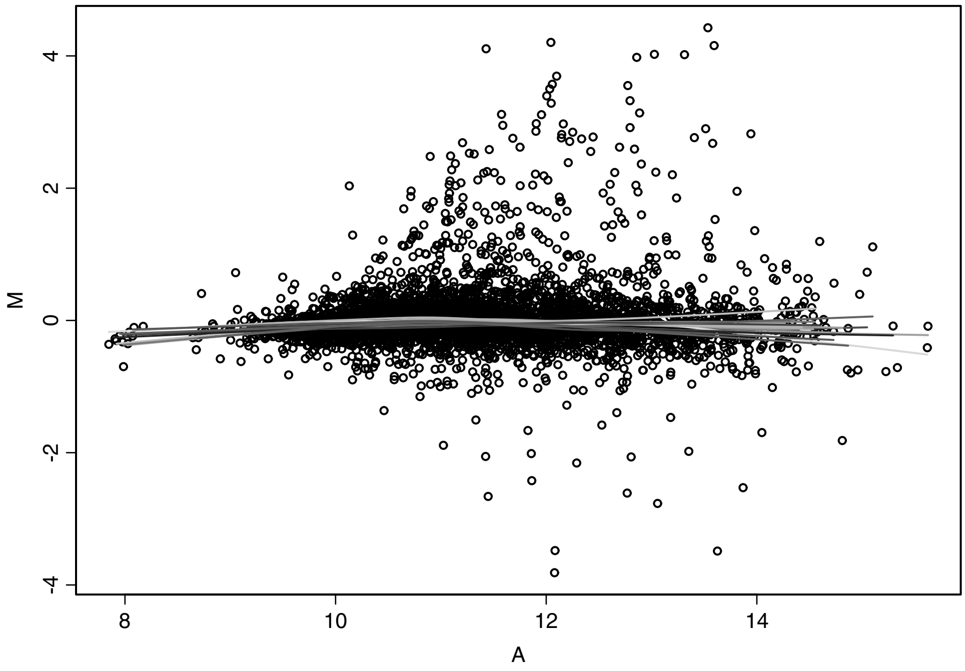

**Figure 6.** For Lrp expression data: $M$ vs. $A$ plot with 16 individual lowess smoothing lines superimposed, one for each print-tip group in slide 1

Copyright © 2004 John Wiley & Sons, Ltd.

normalization methods were developed to reduce the systematic biases and variations across microarray experiments. Before doing any data analysis, we must decide whether we need to normalize the data, and if so which normalization method to use. Here we have illustrated the preliminary use of $M$ vs. $A$ plots, spatial plots and boxplots to decide whether there are any patterns between $M$ and $A$ and whether there are spatial effects. If so, normalization is needed, with the print-tip group normalization method being most appropriate if there is evidence of a within-tip spatial effect. This should also result in a reduction of the false discovery rate in subsequent analysis. For our data, both methodologies (plot inspection and FDR calculation) point to the use of print-tip group normalization being preferable. Hence, we suggest preliminary exploration of the various descriptive plots discussed to guide selection of the appropriate normalization method.

Although this suggestion is practical and easy to use, some concerns about normalization still linger. Because normalization methods assume that the mean log ratio intensity for each slide is close to zero, there should be a small proportion of genes with different intensities. But it can be difficult to check whether a dataset satisfies these assumptions, and whether the normalization methods will still work if the data violate the assumptions. If not, can more robust methods be found? Tseng *et al.* (2001) suggested a rank invariant method and Reilly *et al.* (2003) proposed probability models fit using the Gibbs sampler (see e.g. Carlin and Louis, 2000) to select non-differentially expressed genes to do normalization. ANOVA methods proposed by Kerr *et al.* (2000) are also widely used in normalization and testing for microarray data. One might also compare ANOVA methods with these other methods to see how they perform.

The $B$ statistic uses information from all the genes to estimate the posterior odds of rejecting the null hypothesis. Although the normality and independence assumptions were used to derive the formula for $B$, we did not use a formal test based on these assumptions. Furthermore, the conclusion of 'extremeness' for a gene is often based solely on the rank of its test statistic. Because we cannot estimate the prior probability of extremeness satisfactorily and the scale of $B$ depends on this probability, we cannot use a predetermined cutoff value (such as $B = 0$) for gene selection. Fortunately, the ranks of the various $B$ statistics do not depend on $p$, so we can select the top genes with the most extreme $B$ values based on their ranks. Based on the analysis results for the Lrp data, we conclude that the $B$ statistic performs much better than the $M$ and $t$ statistics, since $B$ yields much smaller false positive rates than the other two. The performance of $B$, in terms of the overlap rate and false discovery rate, is quite similar to that of $S$, but the former is more explicitly model-based. Also, because $B$ has a closed form, it is easy to compute and convenient to use (e.g. it is directly available within the SMA package). Hence, we conclude that, although the $M$ and $t$ statistics are the most commonly used statistics for microarray data analysis, based on our results, we instead recommend the $B$ statistic. It will be interesting to see whether the $B$ statistic can be incorporated into other statistical packages and applications in the detection of differential gene expression (Pan, 2002; Kendziorski *et al.*, 2003; Newton and Kendziorski, 2003).

In this paper, we used the FDR results from the top 150 identified genes to compare the different methods. In order to check whether the results are sensitive to the number of genes so selected, we repeated our analysis using different cut-off points; specifically, using the top 20, 50 and 200 identified genes. The results were consistent with our previous findings: the FDR was highest for non-normalization and lowest for print-tip normalization, and the $B$ and $S$ statistics have lower FDRs than $M$ and $t$. Thus, our results appear robust with respect to the choice of extremeness cut-off point.

Our data analysis followed the common approach of using background subtracted measurement intensity. But Kooperberg *et al.* (2002) pointed out that this method may lead to a much larger variance than needed when the expression levels are low. Qin and Kerr (2003) also showed that background subtraction can increase the variability of gene expression and worsens one's ability to detect the expressed genes. To investigate this issue in our setting, we redid our analyses without background subtraction. Our results were again consistent with our previous conclusions: print-tip normalization and the $B$ statistic enjoy the best FDR performance. We also found that the FDR is consistently lower when using data without background subtraction across different normalization methods and test statistics. Thus, based on our results, avoiding

Copyright © 2004 John Wiley & Sons, Ltd. 

background subtraction seems preferable, but further investigation is needed.

Although there exists some distinction between chromatin immunoprecipitation experiment with genomic DNA as controls and the usual cDNA transcriptional profiling experiment; the copy number of genomic DNA is fixed across the microarray, which is not true for a transcriptional profiling experiment. In our chromatin immunoprecipitation data, we assume that most of genes are not the binding targets of Lrp and, after the transformation (subtracting the median of intensity ratio), the null hypothesis is that the intensity ratio from two channels should be zero. For our transcriptional profiling experiment, we also assume that there is only a small number of genes expressing differently, and the null hypothesis is again that the intensity ratio from two channel should be zero. These common properties between two different types of experiments make us think that similar analysis methods, including normalization and testing methods, may apply to both of these experiments. Our results for both the chromatin immunoprecipitation experiment and the transcriptional profiling experiment are consistent; inspecting the descriptive plots is helpful in choosing the normalization method, and the $B$ statistic is consistently better than the $M$ and $t$ statistics in the sense of lowering the FDR.

### Acknowledgements

We thank Dr Rowena Matthews from the University of Michigan in Ann Arbor for providing us with Lrp antibodies. The work is supported by NIH Grants HL65462 (WP&YX), AI41966 (BPC&YX), GM066098 (AK&KSJ) and NSF/EPA Grant SES99-78238 (BPC&YX).

### References

Lackie JM, Dow JAT. 1999. *The Dictionary of Cell and Molecular Biology*, 3rd edn. Academic Press: London.

Brazma A, Robinson A, Cameron G, Ashburner M. 2000. One-stop shop for microarray data. *Nature* **403**: 699–700.

Tani T, Khodursky A, Blumenthal R, Brown P, Matthews R. 2002. Adaptation to famine: a family of stationary-phase genes revealed by microarray analysis. *Proc Natl Acad Sci USA* **99**: 13 471–13 476.

Spellman P, Sherlock G, *et al*. 1998. Comprehensive identification of cell cycle-regulated genes of the yeast *Saccharomyces cerevisiae* by microarray hybridization. *Mol Cell Biol* **9**: 3273–3297.

Ren B, Robert F, *et al*. 2000. Genome-wide location and function of DNA binding proteins. *Science* **290**: 2306–2309.

Iyer V, Horak C, Scafe C, *et al*. 2001. Genomic binding sites of the yeast cell-cycle transcription factors SBF and MBF. *Nature* **409**: 533–538.

Yang YH, Dudoit S, Luu P, Speed T. 2002. Normalization for cDNA microarray data. *Nucleic Acids Res* **30**: e15.

Quackenbush J. 2002. Microarray data normalization and transformation. *Nature Genet Suppl* **32**: 496–501.

West M. 2000. Bayesian regression analysis in the *large p*, *small n* paradigm. Working Paper 00-22. Institute of Statistics and Decision Sciences: Duke University, USA.

Efron B, Morris C. 1973. Combining possibly related estimation problems. *J R Statist Soc B* **35**: 379–421.

Carlin BP, Louis TA. 2000. *Bayes and Empirical Bayes Methods for Data Analysis*, 2nd edn. Chapman and Hall/CRC Press: Boca Raton, FL.

Newton M, Kendziorski C, Richmond C, Blattner F, Tsui K. 2001. On differential variability of expression ratios: improving statistical inference about gene expression changes from microarray data. *J Comput Biol* **8**: 37–52.

Lonnstedt I, Speed T. 2002. Replicated microarray data. *Statist Sinica* **12**: 31–46.

Kendziorski C, Newton M, Lan H, Gould M. 2003. On parametric empirical Bayes methods for comparing multiple groups using replicated gene expression profiles. *Statist Med* **22**: 3899–3914.

Newton M, Kendziorski C. 2003. Parametric empirical Bayes methods for microarrays. In *The Analysis of Gene Expression Data: Methods and Software*, Parmigiani G, Garrett E, Irizarry R, Zeger S (eds). Springer-Verlag: New York.

Lin Y, Nadler S, Lan H, Attie A, Yandell B. 2003. Adaptive gene picking with microarray data: detecting important low abundance signals. *The Analysis of Gene Expression Data: Methods and Software*, Parmigiani G, Garrett E, Irizarry R, Zeger S (eds). Springer-Verlag: New York.

Newton M, Noueiry A, Sarkar D, Ahlquist P. 2004. Detecting differential gene expression with a semiparametric hierarchical mixture method. *Biostatistics* **5**: 155–176.

Irizarry R, Hobbs B, Collin F, *et al*. 2003. Exploration, normalization, and summaries of high density oligonucleotide array probe level data. *Biostatistics* **4**: 249–264.

Tusher V, Tibshirani R, Chu G. 2001. Significance analysis of microarrays applied to the ionizing radiation response. *Proc Natl Acad Sci USA* **98**: 5116–5121.

Baldi P, Long AD. 2001. A Bayesian framework for the analysis of microarray expression data: regularized $t$-test and statistical inferences of gene changes. *Bioinformatics* **17**: 509–519.

Efron B, Tibshirani R, Storey JD, Tusher V. 2001. Empirical Bayes analysis of a microarray experiment. *J Am Statist Assoc* **96**: 1151–1160.

Benjamini Y, Hochberg Y. 1995. Controlling the false discovery rate: a practical and powerful approach to multiple testing. *J R Statist Soc B* **57**: 289–300.

Dudoit S, Yang YH, Speed T. 2002. The SMA package. Available online at: **www.stat.berkeley.edu/users/terry/zarray/Software/smacode.html**.

Pan W. 2003. On the use of permutation in and the performance of a class of nonparametric methods to detect differential gene expression. *Bioinformatics* **19**: 1333–1340.

Storey J, Tibshirani R. 2003. Statistical significance for genome-wide studies. *Proc Natl Acad Sci USA* **100**: 9440–9445.

Copyright © 2004 John Wiley & Sons, Ltd. 

Efron B, Tibshirani R, Goss V, Chu G. 2000. Microarrays and their use in a comparative experiment. Technical report, Department of Statistics, Stanford University, USA.

Tseng G, Oh M, Rohlin L, Liao J, Wong W. 2001. Issues in cDNA microarray analysis: quality filtering, channel normalization, models of variations and assessment of gene effects. *Nucleic Acids Res* **29**: 3549–2557.

Reilly C, Wang C, Rutherford M. 2003. A method for normalizing microarrays using the genes that are not differentially expressed. *J Am Statist Assoc* **98**: 868–878.

Kerr MK, Martin M, Churchill GA. 2000. Analysis of variance for gene expression microarrays. *J Comput Biol* **7**: 819–837.

Pan W. 2002. A comparative review of statistical methods for discovering differentially expressed genes in replicated microarray experiments. *Bioinformatics* **12**: 546–554.

Kooperberg C, Fazzio T, Delrow J, Tsukiyama T. 2002. Improved background correction for spotted DNA microarrays. *J Comput Biol* **9**: 55–66.

Qin L, Kerr K. 2003. Empirical evaluation of methodologies for microarray data analysis. Available online at: **www.ima.umn.edu/talks/workshops/9-29-10-3.2003/kerrIMA.pdf**.

Copyright © 2004 John Wiley & Sons, Ltd. 
